# Quality of Care after Acute Coronary Syndromes in a Prospective Cohort with Reasons for Non-Prescription of Recommended Medications

**DOI:** 10.1371/journal.pone.0093147

**Published:** 2014-03-27

**Authors:** Reto Auer, Baris Gencer, Lorenz Räber, Roland Klingenberg, Sebastian Carballo, David Carballo, David Nanchen, Jacques Cornuz, John-Paul Vader, Pierre Vogt, Peter Jüni, Christian M. Matter, Stephan Windecker, Thomas Felix Lüscher, François Mach, Nicolas Rodondi

**Affiliations:** 1 Department of Epidemiology and Biostatistics, UCSF, San Francisco, California, United States of America; 2 Division of Cardiology, Faculty of Medicine, Geneva University Hospitals, Geneva, Switzerland; 3 Department of Cardiology, University Hospital Bern, Bern, Switzerland; 4 Department of Cardiology, University Hospital Zurich, Zurich, Switzerland; 5 Division of Internal Medicine, Faculty of Medicine, Geneva University Hospitals, Geneva, Switzerland; 6 Department of Ambulatory and Community Medicine, University of Lausanne, Lausanne, Switzerland; 7 Institute of Social and Preventive Medicine, University of Lausanne, Lausanne, Switzerland; 8 Department of Cardiology, Lausanne University Hospital, Lausanne, Switzerland; 9 Institute of Social and Preventive Medicine and Clinical Trials Unit, Department of Clinical Research, University of Bern, Bern, Switzerland; 10 Department of General Internal Medicine, University Hospital of Bern, Bern, Switzerland; University of Bologna, Italy

## Abstract

**Background:**

Adherence to guidelines is associated with improved outcomes of patients with acute coronary syndrome (ACS). Clinical registries developed to assess quality of care at discharge often do not collect the reasons for non-prescription for proven efficacious preventive medication in Continental Europe. In a prospective cohort of patients hospitalized for an ACS, we aimed at measuring the rate of recommended treatment at discharge, using pre-specified quality indicators recommended in cardiologic guidelines and including systematic collection of reasons for non-prescription for preventive medications.

**Methods:**

In a prospective cohort with 1260 patients hospitalized for ACS, we measured the rate of recommended treatment at discharge in 4 academic centers in Switzerland. Performance measures for medication at discharge were pre-specified according to guidelines, systematically collected for all patients and included in a centralized database.

**Results:**

Six hundred and eighty eight patients(54.6%) were discharged with a main diagnosis of STEMI, 491(39%) of NSTEMI and 81(6.4%) of unstable angina. Mean age was 64 years and 21.3% were women. 94.6% were prescribed angiotensin converting enzyme inhibitors/angiotensin II receptor blockers at discharge when only considering raw prescription rates, but increased to 99.5% when including reasons non-prescription. For statins, rates increased from 98% to 98.6% when including reasons for non-prescription and for beta-blockers, from 82% to 93%. For aspirin, rates further increased from 99.4% to 100% and from to 99.8% to 100% for P2Y12 inhibitors.

**Conclusions:**

We found a very high adherence to ACS guidelines for drug prescriptions at discharge when including reasons for non-prescription to drug therapy. For beta-blockers, prescription rates were suboptimal, even after taking into account reason for non-prescription. In an era of improving quality of care to achieve 100% prescription rates at discharge unless contra-indicated, pre-specification of reasons for non-prescription for cardiovascular preventive medication permits to identify remaining gaps in quality of care at discharge.

**Trial Registration:**

ClinicalTrials.gov NCT01000701

## Introduction

Cardiovascular disease remains the leading cause of death in adults in the United States (US) and in Europe. Acute coronary syndrome (ACS) is the most frequent cause leading to myocardial infarction, heart failure, and sudden death [1]. In-hospital initiation of evidence-based cardiovascular medication has been shown to improve long-term drug adherence and clinical outcomes [2], [3], [4].

Systematic monitoring of performance and annual report cards on quality of care, such as the US Healthcare Effectiveness Data and Information Set (HEDIS) [5], and financial incentives to improve quality are not implemented in Switzerland. Current clinical registries such as the NCDR ACTION Registry-GWTG (National Cardiovascular Data Registry (NCDR) ACC's Acute Coronary Treatment and Intervention Outcomes (ACTION) Registry- Get With the Guidelines (GWTG)) Network, a voluntary participation registry of patients admitted with ACS in the USA, the data collection to determine the rate of prescription of recommended treatment at discharge includes a box to systematically measure if the treatment was contraindicated [6]. Current clinical registries in Europe such as the FAST-MI registry [7], [8], or the APTOR registry [9], do not collect the reasons for non-prescription. A recent report on quality at discharge in Switzerland for patients discharged after a ST-elevation myocardial infarction (STEMI) has shown an improvement in quality of care over the last 15 years, but still suboptimal prescription rates of recommended therapies at discharge [10], [11], [12]. However, given that reasons for non-prescription were not collected, it is unknown if differences are due to remaining gaps in quality of care of if they are due to the absence of reporting on the reasons for non-prescription.

We aimed at measuring the rate of recommended treatment at discharge for patients hospitalized for an ACS in 4 university hospitals in Switzerland, using pre-specified quality indicator recommended in cardiologic guidelines in a centralized database, and including systematic collection of reason for non-prescription for preventive medication.

## Methods

### Study setting and participants

The SPUM-ACS (Special Program University Medicine-Acute Coronary Syndromes) research network was established in 2008 and collects data since 2009 on a prospective cohort of patients hospitalized for an ACS in 4 university medical centers in Switzerland (University hospital of Bern (BE), Geneva (GE), Lausanne (LA) and Zürich (ZH)) [13], [14]. We prospectively included patients hospitalized from September 2009 to October 2010, aged >18 years, hospitalized within 72 hours after pain onset with the main diagnosis of ACS. ACS was defined as patients with symptoms comparable with angina pectoris (chest pain, dyspnea) and at least one of the following characteristics: ST-segment elevation or depression, T inversion or dynamic ECG changes, evidence of positive Troponin and known coronary heart disease (status after myocardial infarction, bypass surgery or PTCA) [15]. The final ACS diagnosis was classified as follows: STEMI (ST-segment elevation myocardial infarction or NSTEMI non ST-segment elevation myocardial infarction or unstable angina. Patients were included in the catheterization laboratory in two participating hospitals (ZH and BE) and additionally while on ward in two participating hospitals (LA and GE). In order to allow comparison with other databases [6], [16], we report on data of patients who were discharged alive from each hospital.

### Ethics statement

The study protocol was approved by the institutional review board of all participating centers; namely, the Ethics Committee on Clinical Research of the University of Lausanne, the Ethics Committee of the Department for Internal Medicine and Community Medicine of the University Hospital of Geneva, the Cantonal Ethics Committee (KEK) of the Canton of Bern, and the Cantonal Ethics Committee (KEK) of the Canton of Zurich. All patients provided written, informed consent.

### Data collection and endpoints

Clinical data for the patients included in the SPUM-ACS study were collected by trained nurses and medical doctors on standardized, web-based case report forms and stored in a central database (Cardiobase, Clinical Trial Unit, and Department of Cardiology, Bern University Hospital, Switzerland, and 2 mT, Ulm, Germany). Data abstracted were (1) demographic characteristics such as age, sex, race, education and (2) medical history, such as previous coronary heart disease (CHD), stroke, renal failure requiring dialysis, valvular heart disease, congestive heart failure (CHF) as well as hypercholesterolemia, hypertension and diabetes. We further collected administrative data of the hospital stay (length of stay, direct transfer to a peripheral hospital or to inpatient cardiac rehabilitation) and prescription of recommended medication at discharge such as aspirin, P2Y12 inhibitors (clopidogrel, prasugrel or ticagrelor), beta-blockers, angiotensin converting enzyme inhibitors/angiotensin II receptor blockers (ACEI/ATII) and statins.

### Performance measures

Performance measures for medication at discharge were pre-specified, systematically collected for all patients and included in the centralized database. They were based on the ACC/AHA 2008 performance measures for adults with STEMI and NSTEMI and included the following pre-specified reasons for non-prescription [17]: for all medications, the reason “other reason documented by physician” and “patient refusal” were included. The reason in full text was entered when the reason “other reason documented by physician“ was selected. Additionally, pre-specified reasons for aspirin were: “active bleeding during hospital stay”, “coumadin/warfarin prescribed at discharge” and “aspirin allergy”; for ACEI/ATII reasons were: “Moderate or severe aortic stenosis” and “ACEI and/or ATII allergy”; for beta-blockers, reasons were: “beta-blocker allergy” and “second- or third-degree atrio-ventricular heart block”. We also included the reason “Bradycardia (heart rate <60/min) on day of discharge” given the frequency of this reported reason by physicians in some centers and despite its absence of recognition an acceptable reason for non-prescription in guidelines [17]. Patients who had “to be introduced later” as the reason for non-prescription of beta-blockers and who had been discharged home directly, were coded as not having been prescribed the recommended medication. We systematically collected information if patients had been offered a specialized smoking cessation intervention in 2 university hospitals (LA, GE). We did not collect information on brief smoking cessation counseling interventions that may have taken place during the hospital stay. In the US, smoking cessation counseling was systematically monitored as part of a pay-for-performance scheme rewarding hospitals for providing smoking cessation intervention. However, recent analyses in the US documented that hospitals were able to “game the system,” with scores approaching 100% on the tobacco-treatment measure [18], prompting the National Quality Forum to abandon tobacco-use intervention as a quality measure [19].

### Statistical analyses

Frequencies, means with standard deviations (SDs), medians with interquartile ranges (IQR) were used when appropriate, as were chi2 tests, Fisher's exact test, Wilcoxon rank sum test and ANOVA for bivariate analyses. Statistical significance was set at 0.05. All analyses were performed using STATA version 12 (StataCorp, College Station, Texas).

## Results

### Patients characteristics

A total of 1260 patients with a main diagnosis of ACS were discharged from 4 university hospitals from September 2009 to October 2010 (Table 1). 688 patients (54.6%) were discharged with a main diagnosis of STEMI, 491 (39%) of NSTEMI and 81 (6.4%) of unstable angina. Mean age was 64 years and 21.3% were women. 22% had had a previous CHD and 38.2% were current smokers. Median length of stay for patients directly transferred home was 5.5 days for patients with STEMI, 3.7 among participants with NSTEMI and 2 among patients with unstable angina. 541 (43%) were discharged home after the hospital stay, 190 (15%) were directly transferred to a stationary cardiac rehabilitation facility and 529 (42%) to a peripheral hospital (Table 1).

**Table 1:** Baseline characteristics of the participants to the study hospitalized for an acute coronary syndrome in 4 academic centers in Switzerland from September 2009 to October 2010.

	Overall N = 1260	Unstable Angina N = 81	NSTEMI N = 491	STEMI N = 688
**Demographic variables**				
Age, y (mean ± SD)	64±12	67±12	65±13	62±12
- <50 years, N (col. %)	183 (14.5)	7 (8.6)	65 (13.4)	111 (16.3)
- 50 to <65 years, N (col. %)	496 (39.4)	25 (30.9)	181 (36.9)	290 (42.0)
- 65 to 80 years, N (col. %)	438 (34.8)	36 (44.4)	177 (36.1)	225 (32.7)
- >80 years, N (col. %)	143 (11.4)	13 (16.1)	68 (13.9)	62 (9.0)
Female, N (%)	268 (21.3)	16 (19.8)	114 (23.2)	138 (20.1)
Race, N (%)				
- Caucasian	1189 (94.6)	76 (93.8)	461 (94.4)	652 (94.8)
- Black	5 (.4)	0 (.0)	2 (.4)	3 (.4)
- Asian	7 (.6)	2 (2.5)	1 (.2)	4 (.6)
- Other	59 (4.7)	3 (3.7)	27 (5.5)	29 (4.2)
Education*				
- Lower than apprenticeship, N (col. %)	230 (20.4)	21 (26.6)	103 (23.8)	106 (17.2)
- Apprenticeship or vocational school, N (col. %)	599 (53.6)	39 (49.4)	214 (49.5)	346 (56.9)
- High School or university graduation, N (col. %)	300 (26.6)	19 (24.1)	115 (26.6)	166 (26.8)
**Clinical history**				
Previous hypercholesterolemia†, N (%)	742 (58.9)	62 (76.5)	327 (66.7)	353 (51.3)
Previous hypertension†, N (%)	742 (58.9)	62 (76.5)	327 (66.7)	353 (51.3)
Previous diabetes†, N (%)	227 (18.3)	16 (19.8)	109 (22.2)	102 (14.8)
Previous CHD, N (%)	276 (22.0)	51 (63.0)	135 (27.6)	90 (13.1)
- Previous PCI, N (%)	209 (16.6)	42 (51.9)	99 (20.3)	68 (9.9)
- Previous CABG, N (%)	74 (5.9)	15 (18.5)	37 (7.6)	22 (3.2)
Previous stroke, N (%)	36 (2.9)	3 (3.7)	16 (3.4)	17 (2.5)
Previous renal failure requiring dialysis, N (%)	8 (.7)	1 (1.2)	5 (1.1)	2 (.3)
Previous valvular heart disease, N (%)	32 (2.5)	1 (1.2)	23 (4.6)	8 (1.2)
**Anthropomorphic variables**				
Obesity (BMI≥30 kg/m^2^)	268 (21.6)	20 (25)	121 (24.9)	127 (18.8)
**Behavioral variables**				
Current smoker, N (%)	480 (38.2)	23 (28.4)	170 (34.6)	287 (41.9)
**Clinical variables**				
Left ventricular function, mean (± SD)	51.5 (±11.4)	55.7 (±10.1)	54.7 (±11.3)	48.9 (±10.9)
- Left ventricular dysfunction (LVEF≤40%), N (%)	220 (20.1)	6 (9.2)	58 (13.7)	156 (25.7)
**Hospital stay**				
Coronary Revascularization				
- Overall revascularization, N (%)	1170 (92.8)	56 (69.7)	439 (89.4)	675 (98.1)
* PCI, N (%)	1115 (88.4)	52 (64.2)	402 (81.9)	661 (96.1)
* CABG, N (%)	55 (4.4)	4 (4.9)	37 (7.5)	14 (2.0)
Destination at discharge, N (%)				
- Home	541 (42.9)	62 (76.4)	231 (47.1)	248 (36.0)
- Direct transfer to inpatient cardiac rehabilitation	190 (15.1)	6 (7.4)	100 (20.4)	84 (12.2)
- Transfer to peripheral hospital	529 (42.0)	13 (16.1)	160 (32.6)	356 (51.7)
Length of stay, median (Q1,Q3), in days				
- For patients directly discharged home	4.4 (2.3, 7)	2 (1, 5)	3.7 (1.9, 6.1)	5.5 (4.0, 7.2)
- For patients transferred to peripheral hospital	1 (.5, 1.5)	1 (.5, 1.5)	1 (.5, 1.5)	1 (.5, 1.5)

N, number of participants; BMI, body mass index; CABG, coronary artery by-pass graft; CHD, coronary heart disease; CR: cardiac rehabilitation; LVEF: Left ventricular ejection fraction; CHF, congestive heart failure; NSTEMI: Non ST-segment elevation myocardial infarction; PCI, percutaneous coronary intervention; Q1: first quartile; Q3; third quartile; STEMI: ST-segment elevation myocardial infarction.

*38 participants with missing information on education status or who refused to disclose their education status.

† Previous hypercholesterolemia, hypertension and diabetes based on self-report by patients or previous treatment by preventive medication specific to the hypercholesterolemia, hypertension or diabetes.

### Prescription rates at discharge

**Figure 1:**
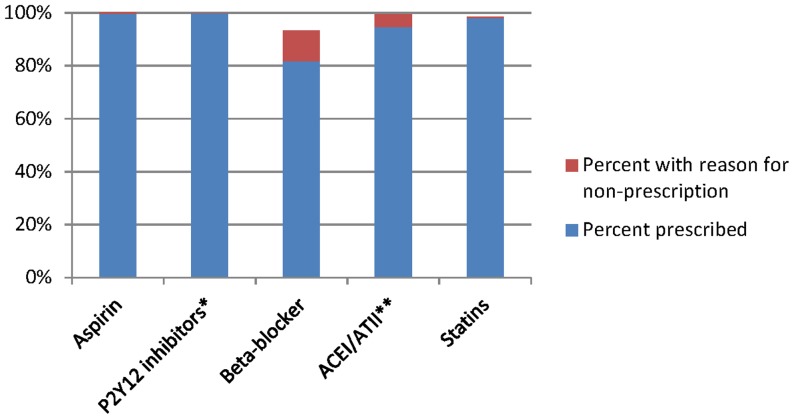
Percent of participants with recommended treatment at discharge taking into account reported reasons for non-prescription. Abbreviations: P2Y12 inhibitors: clopidogrel, prasugrel or ticagrelor; ACEI/ATII : Angiotensin converting enzyme inhibitor/angiotensin II receptor blockers. * P2Y12 inhibitors if PCI-stent treatment (n = 1066). ** ACEI/ATII inhibitors if left ventricular ejection fraction (LVEF) ≤40% (n = 220).

For patients with a left ventricular ejection fraction (LVEF) of ≤40%, the rate of patients hospitalized for ACS who were prescribed ACEI/ATII at discharge was 94.6%. However, when including reasons non-prescription, 99.6% were prescribed ACEI/ATII or had a documented reason for non-prescription at discharge. The rate of patients hospitalized for ACS who were discharged on statins increased from 98% to 98.6% when including reasons for non-prescription. For beta-blockers, rates increased from 82% to 93% and for aspirin, from 99.4% to 100%. For patients that had had a PCI-stent treatment, rates further increase from 99.8% to 100% for P2Y12 inhibitor or had a documented reason for non-prescription and 99.9% on dual antiplatelet therapy (Table 2, Figure 1).147 patients discharged home directly or to another facility had a documented reason for non-prescription for beta-blockers, 8 for aspirin, 11 for ACEI/ATII and 7 for statins (Table 3). The most commonly reported reason for non-prescription for beta blockers was bradycardia (n = 62), defined as a heart rate of <60 beats/minute on the day of discharge. Sixty out of 1260 were discharged without the recommended treatment and without any documented contra-indication. Considering those discharged home directly, the type of ACS was associated with lower rate of treatment at discharge. 7 (11%) participants with UA did not receive the recommended treatment at discharge, 20(9%) with NSTEMI and 7(3%) with STEMI (p = <0.001 for the comparison between STEMI and UA or NSTEMI).

**Table 2:** Documented Treatment at Discharge for participants hospitalized for an acute coronary syndrome in 4 academic centers Switzerland from Sept 2009 to October 2010.

	Overall N = 1,260	Unstable angina N = 81	NSTEMI N = 491	STEMI N = 688
**Aspirin, % including reasons for not prescribing (NP)**	**100%**	**100%**	**100%**	**100%**
- % prescribed regardless of reasons for NP	99.4%	98.8%	99%	99.7%
- Reason for NP documented, N/N eligible	8/1260	1/81	5/491	2/688
**P2Y12 inhibitors if PCI-stent treatment, % including reasons for NP**	**99.9%**	**100%**	**99.7%**	**100%**
- % prescribed regardless of reasons for NP	99.8%	100%	99.5%	100%
- Reason for NP documented, N/N eligible	1/1066	0/47	1/379	0/640
**Dual antiplatelet therapy (DAPT) if PCI-stent treatment, % including reasons for NP**	**99.9%**	**100%**	**99.7%**	**100%**
**Beta-blockers, % including reasons for NP**	**93.3%**	**87.7%**	**90.4%**	**95.9%**
- % prescribed regardless of reasons for NP	81.7%	76.5%	83.3%	81.1%
- Reason for NP documented, N/N eligible	147/1260	11/81	45/491	91/688
**AT II antagonist/ACE inhibitors (LVEF≤40%), % including reasons for NP**	**99.5%**	**83.3%**	**100%**	**100%**
- % prescribed regardless of reasons for NP	94.6%	83.3%	87.9%	97.4%
- Reason for NP documented, N/N eligible	11/220	0/6	7/58	4/156
**Statins, % including reasons for NP**	**98.6%**	**96.3%**	**98.0%**	**99.3%**
- % prescribed regardless of reasons for NP	98.0%	95.1%	97.4%	98.8%
- Reason for NP documented, N/N eligible	7/1260	1/81	3/491	3/688
**Concomitant documentation of Aspirin, Statin, Beta-blockers and AT II antagonist/ACE inhibitors, %** *	**95.2%**	**88.9%**	**94.3%**	**96.7%**
**Nitrate documentation, %**	6.9%	18.5%	9.6%	3.6%

DAPT: Dual antiplatelet therapy; N, number of participants; STEMI: ST-segment elevation myocardial infarction; NP: non-prescription; NSTEMI: Non ST-segment elevation myocardial infarction; LVEF, left ventricular ejection fraction.

* Concomitant prescription at discharge unless contra-indicated or not indicated for aspirin, clopidogrel/prasugrel or ticagrelor if PCI-stent treatment, beta-blocker, statin, ACEI if EF≤40%. When participants transferred to peripheral hospital, beta-blocker and ACEI/ATII coded as not applicable.

Prescription rates according to guidelines taking into account reported indications reasons for not prescribing medication at discharge.

**Table 3:** Documented reasons for not prescribing recommended cardiovascular medication at discharge.

	Documented reasons for not prescribing medication in patients discharged home, in cardiac rehabilitation or to another facility	Documented reasons for not prescribing medication in patients discharged home directly
**Aspirin**	**8**	**3**
- Active bleeding during hospital stay	0	0
- Coumadin/warfarin prescribed at discharge	5	2
- Aspirin allergy	3	1
- Other reason documented by physician for not prescribing	0	0
- Patient refusal	0	0
- Introduced later (in peripheral hospital)	0	*NA*
- Other reason	0	0
**Beta-blocker (only patients not transferred in peripheral hospital considered)**	**147**	**41**
- Beta-blocker allergy	2	2
- Second- or third-degree atrio-ventricular heart block	8	4
- Bradycardia (heart rate <60/min) on day of discharge	62	25
- Hypotension	10	3
- Asthma or COPD	1	1
- Other reason documented by physician for not prescribing	17	6
- Patient refusal	0	0
- Introduced later (in peripheral hospital)	47	*NA* †
- Other reason	0	0
**ACEI/ATII (only patients not transferred in peripheral hospital and with LVEF≤40% considered)**	**11**	**1**
- Moderate or severe aortic stenosis	0	0
- ACEI or ATII allergy	1	0
- Other reason documented by physician for not prescribing	1	0
- Renal failure	4	1
- Hypotension	1	0
- Patient refusal	0	0
- Introduced later (in peripheral hospital)	4	*NA*
- Other reason	0	0
**Statins**	**7**	**2**
- Statin medication allergy	0	0
- Reason documented by physician for not prescribing	3	0
- Statin intolerance	3	2
- Patient refusal	0	0
- Introduced later (in peripheral hospital)	1	*NA*
- Other reason	0	0

ACEI: Angiotensin Converting Enzyme Inhibitor; ATII: Angiotensin II receptor blockers; NA: Not applicable; NR: not reported.

† 6 patients discharged home directly and who had “to be introduced later” as the reason for not prescription were coded as not having been prescribed the recommended medication.

33% of smoking participants were offered a specialized smoking cessation intervention in 2 university hospitals (GE, LA).

## Discussion

We found high adherence to ACS guidelines for drug prescriptions when including reasons for non-prescription to drug therapy. For beta-blockers, prescription rates were suboptimal, even after taking into account reason for non-prescription. In addition, bradycardia was often reported as a reason for non-prescription despite its absence from recommended reasons for non-prescription in guidelines. The prescription rate of recommended treatments were between 100% and 99% for antiplatelet therapy, statin therapy and ACEI/ATII for patients with LVEF≤40% after taking into account pre-specified and documented reason for non-prescription, suggesting that the optimal threshold has been achieved for these medications. Despite the proven benefits of dedicated smoking cessation interventions, only 33% of smokers received such an intervention.

In countries with systematic performance monitoring such as the US, an improvement of the recommended discharge medication has been reported [20]. The NCDR ACTION Registry-GWTG Network showed prescription rates for discharge therapies according to percentiles of performance after exclusion of participants with reasons for non-prescription for each medication. Hospitals in the top 10% of performance achieved prescription rates of 99% for aspirin and beta-blockers, 86% for P2Y12 inhibitors, 93% for ACEI/ATII and 94% for statins [6]. These results suggest that Swiss university hospitals would be within the top 10% hospitals in the US for aspirin, P2Y12 inhibitors, ACEI/ATII and statins. For beta-blockers however, the rate of prescription ranged below the top 10%, even after including documented reasons for non-prescription. Various quality improvement strategies have taken place in Switzerland within the last decade at both regional and national level [21]. However, none of these quality improvement strategies included financial incentives to improve quality of care or public reporting of prescription rate at discharge. The improvement in quality of care at discharge might be due to these quality improvement strategies.

Comparative data on quality of care at discharge taking into account reasons for non-prescription to medication is limited in Switzerland. The only study which considered reasons for non-prescription published so far was a retrospective chart review by trained medical doctors which selected patients hospitalized for a main diagnosis of acute myocardial infarction (AMI) (NSTEMI and STEMI) in three out of the four academic medical centers included in our study (BE, GE, LA) [16]. Patients transferred to another hospital for inpatient care or who expired during the hospital stay were excluded. Criteria for recommended medication at discharge in 1999 were derived from the US Cooperative Cardiovascular Project and adapted to the local context [16], [22]. Statins at discharge and attendance to cardiac rehabilitation were not abstracted. Patients in 1999 had similar baseline characteristics, except a higher age (mean age 68.2 vs. 63.6), higher proportion of women (35% vs. 18%) and previous CHD (36% vs. 22%). Comparing 2009–2010 data to the 1999 data, the prescription rate of patients discharged at home after a NSTEMI or STEMI increased from 91% in 1999 to 100% in 2009–2010 for aspirin and from 81% to 94% for beta-blockers. In patients with a left ventricular ejection fraction (LVEF) of less than 40%, the rates increased from 79% to 100% for ACEI/ATII (p<0.001).

Data from a registry in Switzerland (AMIS Plus) on patients with STEMI suggested that 84.2% were discharged on a P2Y12 inhibitor, 96% on aspirin, 89% on an ACEI/ATII, 91.7% on statins and 79.2% on beta-blockers in 2011, but report on quality data was on a voluntary basis and reasons for non-prescription were not reported [10], [11]. Quality at discharge for patients with STEMI has been reported in France showing high prescription rates of evidence based medication [8]. 95% were discharged on aspirin, 84% on beta-blockers and 75% on ACEI. However, reason for non-prescription to prescription medication was not reported. In an era of targets of prescription rates close to 100% unless contra-indicated, pre-specification of the reasons for non-prescription within the data collection forms permits to identify the remaining gaps in quality at discharge. The 100% prescription rates observed for aspirin and P2Y12 inhibitor obviously suggest that no further increase in quality can be achieved for the prescription rates of these medications, however, it permitted us to identify that beta-blockers might still be underutilized and that reasons for non-prescription such as bradycardia, which is not recognized as a contra-indication by current guidelines needs to be improved.

ESC and AHA Guidelines recommend the adoption of dedicated smoking cessation program in each hospital [23], [24]. Only 33% of smokers received dedicated smoking cessation interventions during the hospital stay. The beneficial effect of a systematic high intensity smoking cessation intervention to all smokers is currently assessed in the participating hospitals.

### Potential limitations

These data are derived from university hospitals and might not represent patients hospitalized for an ACS in other hospital in Switzerland. Compared to the AMIS+, a national registry in academic and non-academic centers accounting for 78 out of 106 hospitals treating ACS in Switzerland, the mean age of participants with STEMI was similar in both men and women, but was lower than in other registries in Europe, and rates of revascularization were higher [11], [25], [26]. These differences in mean age and rate of catheterization might be due to the fact that patients were essentially included in the catheterization laboratory in two participating hospitals. Those patients undergoing catheterization are known to be younger than the total number of patients with ACS [27]. Elderly ACS patients and those not undergoing catheterization have been shown to be less likely to receive evidence-based therapies [27], [28]. In these patients, who may have more comorbidities, the adherence to guidelines could be still suboptimal even including the reasons for not prescription. We urge for careful comparison of the reported rates of treatment according to guidelines at discharge in our studied sample with other registries. The aim of this study was not to report on the quality of care for all patients in Switzerland, but to determine the importance of collecting reasons for non-prescription in databases to detect remaining gaps in quality of care in a sample of participants with high rate of recommended treatment at discharge. We found an association between the type of ACS and prescription of recommended treatment, including reasons for non-prescription. These results should be carefully interpreted due to the low number of participants without recommended treatment at discharge. Rates of referral to stationary cardiac rehabilitation were based on information at discharge. In Switzerland, both stationary cardiac rehabilitation and ambulatory cardiac rehabilitation are available. The reported rate of direct transfer to and stationary cardiac rehabilitation facility from the hospital does not reflect the overall attendance rate to cardiac rehabilitation in Switzerland. Future analyses should explore the attendance rate to cardiac rehabilitation using data at discharge and follow-up.

## Conclusions

We found a found high adherence to ACS guidelines for drug prescriptions when including reasons for non-prescription to drug therapy. Achieved rates of prescribed medication at discharge were above 99% for Aspirin, P2Y12 inhibitors, ACEI/ATII and statins. Prescription rates for beta-blockers taking into account reasons for non-prescription were lower at 94%. In an era of improving quality of care to achieve 100% prescription rates at discharge unless contra-indicated, pre-specification of reasons for non-prescription for cardiovascular preventive medication within clinical registries permits to identify remaining gaps in quality of care at discharge.
